# Can the Cambered Bar Enhance Acute Performance in the Bench Press Exercise?

**DOI:** 10.3389/fphys.2020.577400

**Published:** 2020-10-20

**Authors:** Michal Krzysztofik, Adam Zajac, Piotr Żmijewski, Michal Wilk

**Affiliations:** ^1^Institute of Sport Sciences, The Jerzy Kukuczka Academy of Physical Education in Katowice, Katowice, Poland; ^2^Jozef Pilsudski University of Physical Education in Warsaw, Warsaw, Poland

**Keywords:** resistance training, equipment, power, bar velocity, time under tension, range of motion

## Abstract

The main goal of this study was to assess the impact of the cambered bar (CB) during the bench press exercise on power output and bar velocity when compared to a standard bar (SB). Ten healthy strength-trained men (age = 27.9 ± 3.7 years; body mass = 90.1 ± 12.5 kg; resistance training experience = 6.5 ± 2.7 years; bench press one-repetition maximum (1RM) = 118.5 ± 21 kg) performed a single set of 3 repetitions of the bench press exercise with an SB and a CB at 50%1RM to assess differences in peak power output (PP), mean power output (MP), peak bar velocity (PV), and mean bar velocity (MV), range of motion (ROM), and positive work time under load (TUL) between conditions. The *t*-test indicated significantly higher mean ROM for the cambered bar in comparison to the standard bar (52.7 vs. 44.9 cm; *P* < 0.01; ES = 1.40). Further, there was a significantly higher PP (907 vs. 817 W; *P* < 0.01; ES = 0.35), MP (556 vs. 496 W; *P* < 0.01; ES = 0.46), PV (1.24 vs. 1.14 m/s; *P* < 0.01; ES = 0.35) and MV (0.89 vs. 0.82 m/s; *P* < 0.01; ES = 0.34) for the CB condition when compared to the SB. A significantly longer TUL for the CB was observed, when compared to the SB (1.89 vs. 1.51 s; *P* < 0.01; ES = 1.38). The results of this study showed that the CB significantly increased power output and bar velocity in the bench press exercise at 50%1RM compared to the SB. Therefore, the additional ROM, made possible through the use of the CB, allows for the acceleration of the bar through a significantly longer displacement, which has a positive impact on power output. However, a simultaneous increase in TUL may cause higher fatigue when the bench press is performed with the CB compared to the SB.

## Introduction

The bench press is one of the most common resistance exercises used for the development of upper-body strength and power, as well as for complex sports performance (Gorostiaga et al., 2006; García-Pallarés et al., 2011; Stastny et al., 2017; Ortega-Becerra et al., 2018; Maszczyk et al., 2020). Strength-trained athletes often integrate variations of the bench press such as different grip widths and bench positions (incline/decline) (Saeterbakken et al., 2017; Wilk et al., 2019) to alter the movement patterns and muscular requirements as an additional stimulus to break through plateaus and to prevent training monotony (Krzysztofik et al., 2019).

The proper bench press technique relies on lowering the bar to the chest and then pushing it against the direction of gravity until the full extension of the elbows (Gomo and Van Den Tillaar, 2016). When performing the bench press with a standard type bar (SB), the range of motion (ROM) is restricted by the bar which touches the chest, while the prime movers are clearly not going through their full physiological ROM. To overcome this restriction, and increase the ROM during the bench press movement, a cambered type bar (CB) has been introduced. The CB has a U-shape, which creates additional space for the torso, enabling the movement to a lower end position compared to the SB (Krzysztofik et al., 2020a). One of the premises of the CB is to enable the athlete greater stretch of the chest and shoulder muscles in the bottom phase of the movement (Corey, 1991). However, it should be taken into account that the increased ROM may strain the chest and shoulder muscles, especially when the athlete is unfamiliar with a CB bench press. Therefore, for safety reasons, the negative work of CB bench press should be performed in a controlled manner. In summary, the use of a CB bar during the bench press increases the distance the bar needs to be lifted, which potentially may impact exercise performance.

However, only one previous study has analyzed the impact of the CB on muscle performance (Krzysztofik et al., 2020a). The results of the study by Krzysztofik et al. (2020a) indicated greater ROM, as well as different muscle activity during the CB bench press in comparison with the SB. Although, that authors suggested that the implementation of the CB bench press may be beneficial for athletes, especially those whose sports discipline requires a variety of upper limb sports movements (Krzysztofik et al., 2020a) it still many science aspects related with the CB have not been studied. For example, to date, there is no information related to the influence of CB during bench press on the upper-body muscular strength and hypertrophy changes after long-term resistance training or direct comparison of CB and SB on acute power output and bar velocity.

One of the basic differentiating factors during the bench press exercise with the CB and SB is the ROM. Previous studies showed that changes in the ROM impact on acute responses as well as chronic adaptive changes (Bloomquist et al., 2013; Pallarés et al., 2020; Schoenfeld and Grgic, 2020). The changes in the ROM during long-term resistance training impact movement velocity and power output, as well as the utilization of elastic energy derived in the stretch – shortening cycle (Martínez-Cava et al., 2019a; Pallarés et al., 2020). The stretch – shortening cycle is a basic muscle function, where the pre-activated muscle is first stretched and immediately followed by the shortening contraction or may be shortly delayed by a brief transition phase. It has been confirmed that a movement performed after a pre-stretch generates greater power output compared with the shortening – only contraction movements. Furthermore, it has been established that the acute increase of performance due to the stretch – shortening cycle is dependent on the stretch amplitude (Cronin et al., 2001). However, when the stretch phase reaches a critical threshold, the subsequent shortening contraction shows no further increase in power output or may even result in its decrease (Turner and Jeffreys, 2010). In regards to the long-term adaptations, Pallarés et al. (2020) showed that 10 weeks of resistance training with full ROM in the squat exercise led to greater improvements in jump height, as compared to partial ROM in that exercise. Similarly, a study by Martínez-Cava et al. (2019a) showed that 10 weeks of bench press training with full ROM produced greater improvements in maximum strength compared to bench press performed with partial ROM. Furthermore, the extended ROM during a lift may also impact acute changes in bar velocity and power output due to the achievement of longer propulsive phases. Studies by Martínez-Cava et al. (2019b, c) showed that the mean velocity attained against a wide range of loads were significantly higher, with a greater ROM during resistance exercises. Therefore, the CB during the bench press exercise could allow the bar to be accelerated through a greater ROM, which might result in significantly higher bar velocity and power output compared to an SB. Furthermore, the changes in ROM during the resistance exercise can also impact the time under tension or recently used – term time under load (TUL) (Grgic et al., 2018) in a particular set, as well as in a whole training session. A common method of measuring resistance training volume is multiplying the repetitions with the external load (Padulo et al., 2015). However, given the differences in ROM between SB and CB bench press, the comparison of resistance training volume based on repetition or tonnage should be extended to measure of TUL. According to McBride et al. (2009) and Wilk et al. (2020c), the TUL is an indicator of training volume, therefore the changes in ROM can potentially impact the volume effort. However, currently, there is no scientific data available analyzing the acute impact of different ROM on power output and bar velocity during the bench press exercise with a simultaneous analysis of TUL.

Given the widespread use of bench press exercise as a means to develop power output in the upper limbs, the CB can significantly modify and increase the effectiveness of this exercise due to differences in ROM and muscle activity. Further, the CB during the bench press may potentially impact the kinematic variables, what can be of great practical significance, especially to athletes and coaches. Therefore, the main goal of this study was to assess the impact of the CB during the bench press exercise on power output and bar velocity when compared to the SB. Considering the results of previous studies, we hypothesized that the bench press performed with the CB through a greater ROM would enhance acute power output and bar velocity.

## Materials and Methods

### Participants

Ten healthy strength-trained men participated in this study (age = 27.9 ± 3.7 years, body mass = 90.1 ± 12.5 kg), with a minimum of 3 years of resistance training experience (6.5 ± 2.7 years). The inclusion criteria was a bench press personal record of at least 120% of body mass (BP 1RM = 118.5 ± 21 kg) (Krzysztofik et al., 2020b). The study participants were allowed to withdraw from the experiment at any moment and were free from musculoskeletal disorders. They were informed about the benefits and potential risks of the study before providing their written informed consent for participation. The study protocol was approved by the Bioethics Committee for Scientific Research, at the Academy of Physical Education in Katowice, Poland (10/2018), and performed according to the ethical standards of the Declaration of Helsinki, 2013.

### Study Design and Procedure

**FIGURE 1:**
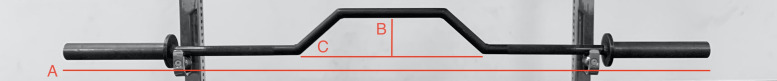
The cambered bar characteristics. Weight – 20 kg; **(A)** overall length – 190 cm; **(B)** camber depth – 10 cm; **(C)** space between camber – 55 cm.

**FIGURE 2:**
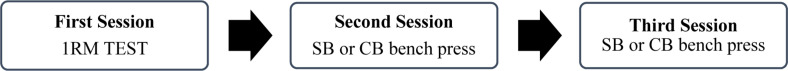
Schematic representation of the experimental sessions protocol.

To familiarize the participants with the CB bench press exercise, all participants underwent familiarization sessions once a week for 2 weeks before starting the experimental sessions. The CB features are presented in the Figure 1. Next, the participants attended three experimental sessions, which were conducted at the same time of the day to avoid circadian variation (in the morning between 8:00 and 10:00 am), and were separated by a 1-week interval. The first session was used to determine the one-repetition maximum (1RM) load of the flat bench press with the SB. The second and the third session was performed in a random order and consisted of performing the bench press exercise with the CB or SB at 50%1RM to record the peak power output (PP), mean power output (MP), peak bar velocity (PV), and mean bar velocity (MV) (Figure 2). The participants were instructed to not perform any additional resistance exercises within 72 h of testing to avoid fatigue. Moreover, they were asked to maintain their normal dietary and sleep habits throughout the study and not to use any supplements or stimulants for 24 h prior to the sessions.

### Standard Bar One-Repetition Maximum Strength Test

A standardized warm-up protocol was used for each session, including a general warm-up of approximately 5 min using a hand cycle ergometer (around 70% of heart rate maximum which corresponds to approximately 130 bpm), followed by a general upper-body warm-up of 10 trunk rotations and trunk side-bends on each side, 10 internal and external rotary movements of the shoulders and 10 push-ups. During the 1RM test, the participants performed 15, 10, and 5 bench press repetitions using 20, 40, and 60% of their estimated 1RM (self-reported by participants before the 1RM test). The first test load was set to an estimated 80%1RM and was increased by 2.5–10 kg for each subsequent attempt. This process was repeated until volitional failure occurs during positive work. During the 1RM test, the participants executed one repetition with a 5 min rest interval between successful trials. According to Wilk et al. (2020a, b) all trials during the 1RM test were performed with a constant duration in the negative work (2 s) and volitional speed in the positive work. Hand placement on the bar was set at 150% of the individual bi-acromial distance and was recorded to ensure consistent hand placement during all experimental sessions. All repetitions were performed without bouncing the bar off the chest, without intentionally pausing at the transition between the negative and positive work, and without raising the hips off the bench. The 1RM test has good-to-excellent test–retest reliability (Grgic et al., 2020).

### Experimental Sessions

The general warm-up for the experimental session was identical to the one used during the 1RM test. The experimental sessions consisted of performing a single set of 3 repetitions of the bench press exercise with a SB or CB at 50%1RM. The participants were asked to perform negative and positive work of the movement with maximal possible velocity. Every repetition was performed without bouncing the bar off the chest, and without intentionally pausing at the transition between the negative and positive work. Hand placement on both bars was measured to ensure consistent during all attempts (150% of the individual bi-acromial distance). All repetitions were directly supervised by an experienced strength and conditioning trainer and performed without bouncing the bar off the chest, deliberately pausing in the transition between the negative and positive work, and without lifting the hips off the bench. No weight-lifting belts, shoes, or other supportive garments were allowed.

A linear position transducer system (Tendo Power Analyzer, Tendo Sport Machines, Trencin, Slovakia) was used for the evaluation of power output and bar velocity, ROM, and positive work TUL during the bench press exercise. The system consists of a velocity sensor connected to the bar with a kevlar cable, which, through the interface, immediately transmits the vertical velocity reached by the bar to special software installed on the computer. The sampling rate is determined by the velocity of the disk’s rotation. In previous studies, this linear transducer has emerged as a reliable system for measuring bar velocity and power output during bench press exercises (intra-class correlation coefficient ∼0.905–0.989) (García-Ramos et al., 2018; Goldsmith et al., 2019). The PP and PV were obtained from the best repetition performed in a particular set. MP, as well as MV, were obtained as the mean of all repetitions performed in particular set.

### Statistical Analysis

The statistical analysis was performed with Statistica 9.1 (Hillview, Palo Alto, CA, United States) and presented as means and standard deviations. To assess differences in the ROM between the standard and cambered bar, as well as the difference in PP, MP, PV, MV and TUL the paired-samples *t*-test was performed. All variables presented a normal distribution according to the Shapiro-Wilk test. Effect sizes (Cohen’s *d*) were reported where appropriate, and interpreted as large (*d* ≥ 0.80); moderate (*d* between 0.79 and 0.50); small (*d* between 0.49 and 0.20); and trivial (*d* < 0.20) (Cohen, 2013). The statistical significance was set at *P* < 0.05.

## Results

The *t*-test indicated significantly higher mean ROM for the CB in comparison to the SB (52.7 vs. 44.9 cm; *P* < 0.01; ES = 1.40). Further, there was a significantly higher PP (907 vs. 817 W; *P* < 0.01; ES = 0.35), MP (556 vs. 496 W; *P* < 0.01; ES = 0.46), PV (1.24 vs. 1.14 m/s; *P* < 0.01; ES = 0.35) and MV (0.89 vs. 0.82 m/s; *P* < 0.01; ES = 0.34) for the CB condition when compared to the SB. A significantly longer TUL for the CB was observed, when compared to the SB (1.89 vs. 1.51 s; *P* < 0.01; ES = 1.38) (Table 1).

**TABLE 1:** Power output and bar velocity measured during the bench press exercise with the standard and cambered bar.

**Variable**	**Standard bar**	**Cambered bar**	**Mean differences**	**95% CI for difference**	**ES**	***P***
Range of motion (cm)	44.84.5	52.36.1	7.5	6.09–8.81	1.40	0.01*
Peak power output (W)	817270	907238	90	148–32	0.35	0.01*
Mean power output (W)	496131	556131	60	37–83	0.46	0.01*
Peak velocity (m/s)	1.140.28	1.240.29	0.10	0.14–0.05	0.35	0.01*
Mean velocity (m/s)	0.820.20	0.890.21	0.07	0.04–0.09	0.34	0.01*
Positive work time under load (s)	1.510.19	1.890.34	0.38	0.47–0.28	1.38	0.01*

Mean ± standard deviation (SD).*Statistically significant difference *P* < 0.05.ES, effect size; CI, confidence interval.

## Discussion

The main finding of the study was that the CB used during the bench press exercise significantly increases bar velocity, and power output compared to the SB reaching a small effect size. Therefore, the use of the CB can be an additional tool for increasing performance of the upper limbs during the bench press exercise. Furthermore, the results of the presented study show significantly increased ROM as well as the TUL during the bench press exercise for the CB when compared to the SB, what is consistent with previous findings (Krzysztofik et al., 2020a).

The results of our study indicated that changes in the ROM potentially can be an important factor affecting kinematics of the bench press movement. The bench press with the CB significantly increased the ROM compared to the SB (44.9 vs. 52.7 cm) reaching the large effect size (1.40), which caused a significant increase in power output and bar velocity in both mean and peak values. Although the reported effects of power output and bar velocity changes have been small (from 0.34 to 0.46), it should be taken into account that even a modest increase in performance can affect training adaptation and make the difference to winning in some sports, that require explosive upper-body strength (Pyne et al., 2009; Grgic et al., 2019). The obtained increase in power output and bar velocity were associated with a longer propulsive phase. The acceleration through a greater portion of the movement at a given load results in the production of significantly greater velocities and power output, what was confirmed by Martínez-Cava et al. (2019a). The Martínez-Cava et al. (2019a) investigated the impact of different ROM (full movement vs. two-third vs. one-third) on the bench press exercise performance with a SB, and showed that mean velocity was significantly higher when the greater ROM was applied. The results of the present study are consistent with those obtained by Martínez-Cava et al. (2019a). Thus, as hypothesized, the use of a CB, due to an extended bottom phase of the lift, contributed to higher bar velocity and greater power output in comparison to the SB.

The increase in power output and bar velocity observed during the bench press with the CB can also be related to more efficient utilization of the stretch and shortening cycle. The magnitude of the stretch and speed of movement in the negative work leads to enhancement of performance during the positive work of the movement (Cronin et al., 2001; Wilk et al., 2020c). However, when the amplitude of stretch reaches a critical threshold or is insufficient, the subsequent positive phase may not be enhanced, or may even decrease (Turner and Jeffreys, 2010). Since the use of the CB led to higher bar velocity and greater power output, it can be concluded that the SB provides insufficient pre-stretch and may not allow for optimal utilization of the stretch and shortening cycle. A study by Krzysztofik et al. (2020a) reported that when a CB was used during the bench press exercise, lower muscle activity of the pectoralis major was recorded in comparison to the SB at the same external load. A decrease in muscle activity may indicate that part of the mechanical energy is stored in the elastic components, such as tendons and ligaments. It can be speculated that in the present study the larger pre-stretch due to the use of a CB caused greater storage and release of elastic energy, thus improvements in bench press performance in the positive work of movement. Further, Newton et al. (1997) suggested that the stretch reflex only enhances the early phase of the concentric contraction, and is diminished over the later phase. However, greater acceleration of the load in the early phase of concentric contraction in conjunction with a longer propulsive phase causes an augmentation of performance during the bench press exercise with the CB what may help explain the obtained results.

**FIGURE 3:**
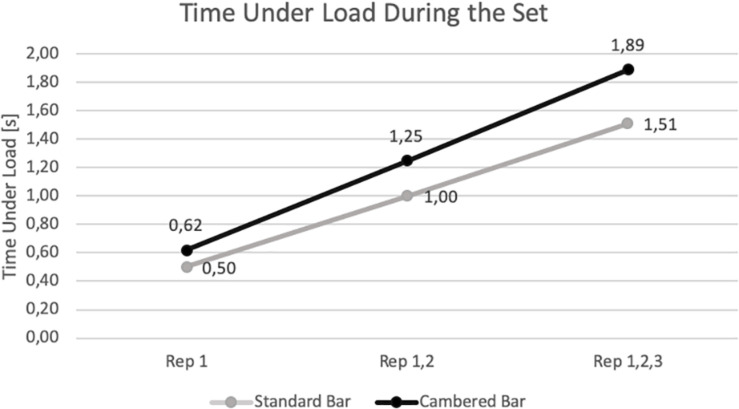
The differences in positive work time under load during subsequent repetitions between the bench press exercise performed with the standard and cambered bar.

However, during the bench press exercise executed with the CB, the improvement of the power output and bar velocity (due to the extended ROM), was accompanied with a significant increase of the TUL in a set, with the large effect size (1.38), when compared to the SB (1.89 vs. 1.51 s respectively). The increase in TUL in the CB due to the extended ROM applies to each repetition performed during a set. Furthermore, the TUL during the bench press exercise with the CB will gradually increase during each subsequent repetition when compared to the SB (Figure 3). Considering that the TUL in the CB will gradually increase during each subsequent repetition, and the higher the number of repetitions, the greater the TUL differences in particular sets between the CB and the SB. According to Wilk et al. (2018, 2020c) and McBride et al. (2009), TUL is one of the indicators of resistance training volume. Longer TUL may affect metabolic stress and endocrine responses during resistance exercise (Wilk et al., 2018, 2020d). Therefore, the lengthening of the ROM and thus an increase in TUL can also increase fatigue, which can negatively affect performance of subsequent repetitions as well as subsequent sets that should be considered when the CB is used during the bench press exercise. Moreover, due to the greater ROM, as well as the longer TUL during exercise with the CB, longer rest intervals may be required between sets compared to the SB, when the training objective is power development. Therefore, when the impact of different ROM on acute responses and chronic adaptation changes is assessed, the time under tension should also be considered.

Although the results of the presented study show an acute increase in power output and bar velocity for the CB compared to the SB, there were also several limitations which should be addressed. The first limitation of the study is the assessment of performance based only on a single value of external load (50%1RM), as well as a single set of the BP exercise. Therefore, the results of the presented study do not translate to other loads as well as higher number or sets. Moreover, the test-retest reliability wasn’t performed, however, the intra-class correlation coefficient of the used linear transducer for measuring bar velocity and power output during bench press exercises is considered as excellent. Further, biomechanical analysis (differences in upper limbs joint angles and moment arms) and electromyography of the lifting technique were not recorded, and no direct physiological variables were assessed which could help explain the obtained results. As the power production and muscular activity patterns relate to sex and training status, the findings of this study should be generalized with caution (Gołaś et al., 2018; Miller et al., 2019). Therefore, future studies should provide a detailed comparison of the velocity-load relationship in the multi-set bench press exercise protocols with a cambered and standard bar, in a large number of experienced strength-trained athletes.

### Practical Implications

The results of this study have important implications for resistance training outcomes. In light of the results of this and prior studies (Krzysztofik et al., 2020a), the bench press exercise performed with the CB seems to be particularly important for sports disciplines, that require the performance of numerous upper-limb explosive movements. Moreover, the additional ROM may elicit not only a greater level of power output but also by the longer TUL can be effective in stimulating muscle strength and hypertrophy. However, the longer TUL can increase the level of fatigue, what should be considered when the CB is used. Nevertheless, strength and conditioning practitioners should bear in mind that the CB used during upper-body resistance exercise demands additional flexibility from athletes and may not be appropriate in athletes after chest and shoulder injuries. Therefore, the introduction of the CB to resistance exercise programs requires a gradual increase of the ROM for athlete’s safety.

## Conclusion

To summarize, a small effect size but a statistically significant increase in power output and bar velocity were observed in the CB bench press exercise at 50%1RM compared to a SB. Therefore, the additional ROM possible through use of the CB, allows for accelerating the bar at a significantly longer distance, what has a positive impact on power output. However, a simultaneous increase in TUL may cause greater fatigue when the bench press is performed with the CB compared to a SB. Thus, it can be assumed, that the CB can be an additional tool to increase the power of the upper limbs, especially useful for athletes representing explosive sports disciplines.

## Data Availability Statement

The raw data supporting the conclusions of this article will be made available by the authors, without undue reservation.

## Ethics Statement

The studies involving human participants were reviewed and approved by the Bioethics Committee at the Academy of Physical Education in Katowice, Poland (10/2018). The patients/participants provided their written informed consent to participate in this study.

## Author Contributions

MK and MW contributed to the conceptualization, data curation, methodology, and writing of the original draft. MW contributed to the formal analysis. MK contributed to the investigation. MW and AZ contributed to the supervision. PŻ and AZ contributed to the writing - review and editing. All authors contributed to the article and approved the submitted version.

## Conflict of Interest

The authors declare that the research was conducted in the absence of any commercial or financial relationships that could be construed as a potential conflict of interest.
